# The Temporal Relationship between Arterial Stiffening and Blood Pressure Is Modified by Methotrexate Treatment in Patients with Rheumatoid Arthritis

**DOI:** 10.3389/fphys.2017.00593

**Published:** 2017-08-15

**Authors:** Richard J. Woodman, Leena R. Baghdadi, Michael E. Shanahan, Arduino A. Mangoni

**Affiliations:** ^1^Centre for Epidemiology and Biostatistics, School of Medicine, Flinders University Adelaide, SA, Australia; ^2^Department of Clinical Pharmacology, School of Medicine, Flinders University and Flinders Medical Centre Adelaide, SA, Australia; ^3^Department of Family and Community Medicine, King Saud University Riyadh, Saudi Arabia; ^4^Department of Rheumatology, Flinders University and Southern Adelaide Local Health Network Adelaide, SA, Australia

**Keywords:** methotrexate, disease-modifying anti-rheumatic drugs, blood pressure, arterial stiffness, pulse wave velocity, rheumatoid arthritis, cross-lagged path analysis

## Abstract

**Background:** The temporal relationship between arterial stiffness and blood pressure (BP) may vary depending on age and other clinical and demographic factors. Since both BP and arterial stiffness are also affected by inflammatory processes, we examined the temporal arterial stiffness-BP relationship in patients with rheumatoid arthritis (RA) treated with either methotrexate (MTX), an anti-rheumatic agent shown to reduce cardiovascular risk in meta-analyses, or other disease-modifying anti-rheumatic drugs (DMARDs).

**Methods:** Measurements of clinic and 24-h peripheral and central systolic and diastolic BP (SBP and DBP), and pulse wave velocity (PWV) were assessed in RA patients on stable treatment with either MTX ± other DMARDs (MTX group, *n* = 41, age 61 ± 14 years, 73% females) or other DMARDs (non-MTX group, *n* = 18, age 65 ± 13 years, 89% females). Measurements were performed at baseline and after 8 months. The temporal relationships were examined using cross-lagged path analysis with models that included age, sex, body mass index, prednisolone, and folic acid use and 28-joint disease activity score.

**Results:** There were significant differences in the temporal arterial stiffness-BP relationships between those in the MTX and DMARD groups. A higher PWV at baseline caused a significant increase in 6 out of 8 different measures of SBP at 8 months amongst those treated with DMARDs (standardized β, range = 0.54–0.66, *p* < 0.003 for each) and 3 out of 8 different measures of DBP (standardized β, range = 0.52–0.61, *p* < 0.003 for each) but was not associated with either SBP or DBP at 8 months amongst those treated with MTX. The difference in the effect of baseline PWV on 8-month BP between the 2 groups was also significant (*p* < 0.003) for 4 measures including clinic peripheral SBP (β = 7.0, 95% CI = 2.8–11.1 mmHg per 1 m/s higher baseline PWV; *p* < 0.001).

**Conclusions:** Higher arterial stiffness preceded increases in BP in subjects with RA treated with DMARDs, but these effects did not occur amongst those treated with MTX. The different effects were seen mostly in measures of SBP but were also present in some measures of DBP. Our findings suggest MTX may confer a protective effect against stiffness mediated increases in BP in patients with RA.

## Introduction

Arterial stiffness and blood pressure (BP) are two well established independent risk factors for cardiovascular disease (CVD), yet both are also closely associated with each other (Mitchell, 2014). The relationship between the two parameters is likely to be bidirectional based on haemodynamic, vascular biology, and physiology principles, with both factors potentially capable of influencing one another. Thus, whilst arterial stiffening may cause increases in blood pressure due to a reduced ability to buffer the BP waveform, it is also possible that the increased damage to arteries from higher BP may lead to an increase in arterial stiffness (Mitchell, 2014). A stronger temporal effect of BP on arterial stiffness than vice-versa was recently observed in middle aged adults without hypertension followed for a period of 7 years (Chen et al., 2016). This was however in contrast to other studies, mostly cross-sectional and in older populations, that have generally found a stronger effect of arterial stiffness on BP (Najjar et al., 2008). The strength of each temporal relationship may therefore depend on the populations studied whereby age and other factors related to the underlying pathophysiology may determine the dominating causal effects. One such factor is represented by chronic systemic inflammation, a common feature in patients with autoimmune disease states such as rheumatoid arthritis (RA), a condition notoriously associated with a significant increase in cardiovascular morbidity and mortality when compared to the general population (Avina-Zubieta et al., 2008).

In addition to their known status as CVD risk factors in the general population, increased BP (Baghdadi et al., 2015) and increased arterial stiffness (Ikdahl et al., 2016) also increase the risk of CVD in RA patients. Methotrexate (MTX) is a commonly used disease-modifying anti-inflammatory drug (DMARD) in this population and observational studies have shown that MTX treatment is associated with a lower clinic BP and a reduced prevalence of hypertension in RA patients (Cuchacovich and Espinoza, 2009; Mangoni et al., 2017). Furthermore, recent meta-analyses have shown that the use of MTX in RA and other chronic inflammatory states is associated with a significantly lower risk of cardiovascular events, including myocardial infarction (Roubille et al., 2015). Since inflammation is also associated with arterial stiffening and elevated BP (Savoia and Schiffrin, 2006; Jain et al., 2014), use of methotrexate may influence the causal effects of elevated stiffness on increases in BP unlike other DMARDs, despite similar anti-inflammatory effects. However, the predominating temporal relationships between BP and arterial stiffness has not yet been assessed in the RA population.

In this medium term follow-up study of 8 months, we performed repeat assessments of arterial stiffness using pulse wave velocity (PWV) and both clinic and 24-h ambulatory blood pressure (peripheral and central) in a cohort of 59 subjects with a confirmed diagnosis of RA. We then determined the temporal associations between stiffness and BP in those subjects treated with methotrexate + DMARDs (*n* = 41) and in those not using methotrexate and treated only with DMARDs (*n* = 18).

## Methods

### Study design

**Figure 1:**
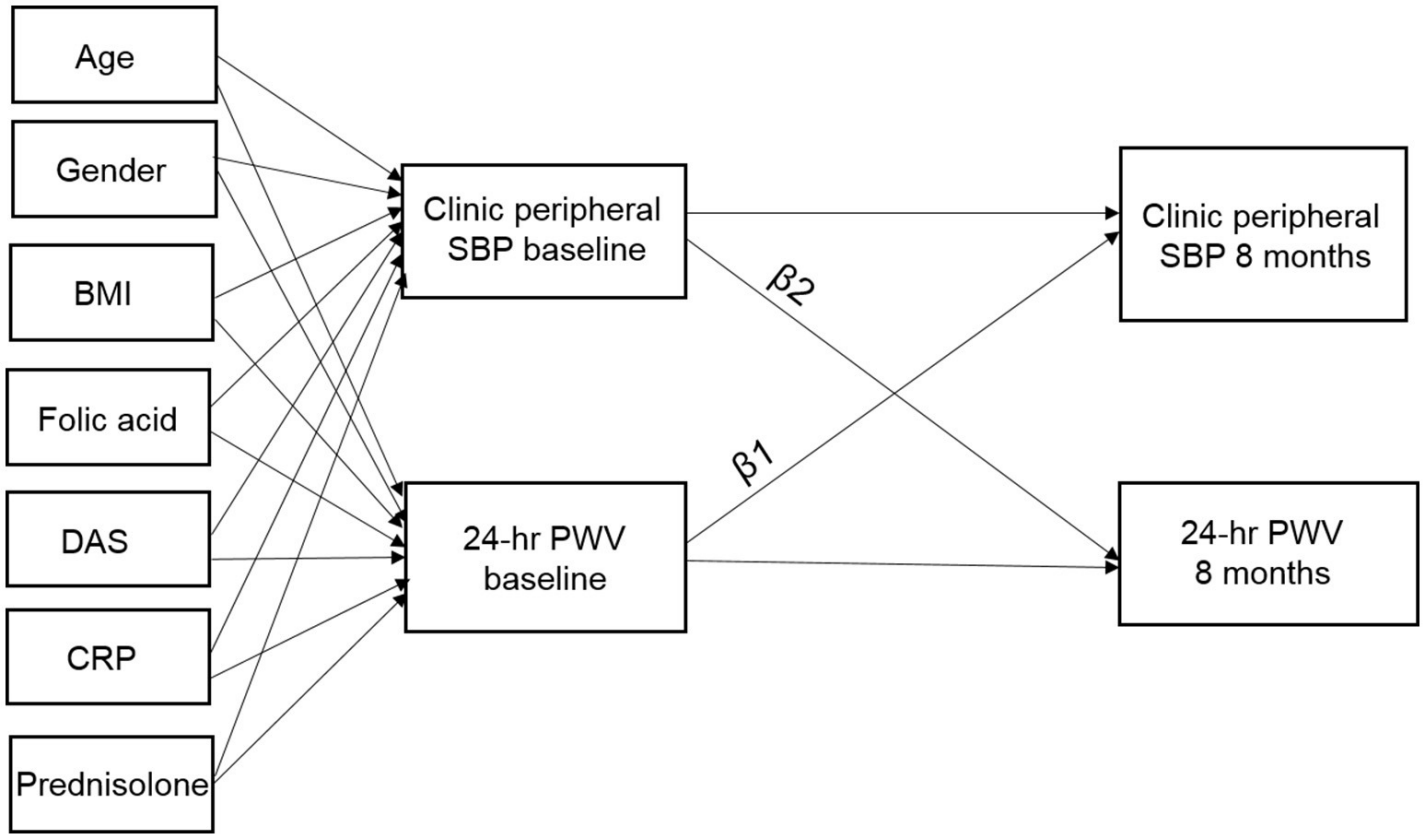
β_1_, cross-lagged path coefficient from baseline PWV to follow-up clinic peripheral systolic blood pressure (SBP); β_2_, cross-lagged path coefficient from baseline clinic peripheral SBP to follow-up PWV. Baseline measures of age, gender, BMI, DAS, serum CRP, and use of folic acid and prednisolone are included as covariates. Arrows indicate hypothesized causal directions and when significant in the cross-lagged path analysis this provides an indication of causality.

We conducted a repeat cross-sectional study with measurements of BP and PWV at baseline and at 8 months follow-up. A cross-lagged panel approach (Selig and Little, 2012) was used for the analysis to determine the strength of the temporal relationships between baseline PWV and 8-month BP, and between baseline BP and 8-month PWV. In total, we recorded 16 measures of BP; 8 × SBP and × DBP, with each including clinic, day-time, night-time, 24-h, for both peripheral and central measures. Figure 1 shows an example of the cross-lagged causal pathways for PWV and clinic peripheral SBP. The arrows indicate causal directions e.g., clinic peripheral SBP at baseline affecting clinic peripheral SBP and PWV at 8-month follow-up. Our primary interest was to determine the strength of the relationships between baseline PWV and follow-up BP (β_1_) and the association between baseline BP and follow-up PWV (β_2_).

### Patient recruitment and ethical approval

We studied a consecutive series of patients with stable RA, aged ≥18 years, recruited from the outpatient clinics of the Rheumatology Department at Flinders Medical Centre and the Repatriation General Hospital, in the Southern Health Local Heath Network, Adelaide, Australia. RA was diagnosed according to the 1987 American College of Rheumatology or the 2010 American College of Rheumatology/European League Against Rheumatism criteria (Aletaha et al., 2010). Study participants were classified as currently treated with MTX for at least 8 weeks (MTX group), or not taking MTX for at least 1 year or being MTX-naïve and treated with other synthetic and/or biologic DMARDs (non-MTX group). Exclusion criteria were atrial fibrillation, active cancer or current treatment with anti-cancer drugs, heart failure, and cognitive impairment.

The study (registered in the Australian New Zealand Clinical Trials Registry with the registration number ACTRN12616001366448) was approved by the Southern Adelaide Clinical Human Research Ethics Committee (Ethics Approval Number: 76.14). Each participant gave written consent before entering the study in accordance with the Declaration of Helsinki.

### Clinic peripheral and central blood pressure

Clinic peripheral systolic (SBP) and diastolic (DBP) BP were measured in the morning, in a quite environment at room temperature, using the clinically validated automatic BP monitor (model UA-767PC; AND Medical, Sydney, Australia) according to current guidelines (Palatini et al., 1998; Baghdadi et al., 2015; Gabb et al., 2016). The average of the last two sitting BP measurements was calculated and used in analyses. Clinic central SBP and DBP was measured non-invasively using Pulse Wave Analysis (PWA, SphygmoCor version 7.1, AtCor Medical, Sydney, Australia; O'Rourke et al., 2001).

### 24-h peripheral and central blood pressure and PWV

Twenty-four hour, daytime (between 08:00 a.m. and 12:00 a.m.), and night-time (between 12:00 a.m. and 08:00 a.m.; Fagard et al., 1996) peripheral and central BP and PWV were measured using a validated ambulatory oscillometric BP monitor (Mobil-O-Graph PWA monitor, IEM, Stolberg, Germany; Jones et al., 2000; Westhoff et al., 2005; Franssen and Imholz, 2010; Wei et al., 2010). The central BP measurement, similar to that measured using the SphygmoCor device (Weiss et al., 2012), is based on the ARCSolver method, which determines aortic BP and PWV using the oscillometric BP technique (Wassertheurer et al., 2010; Weber et al., 2011).

### Clinical and demographic characteristics

The following data were collected from patient interviews, medical questionnaires, clinical notes, and hospital administrative databases: age, gender, medical, and medication history, Stanford health assessment questionnaire (HAQ) (Pincus et al., 1983), pain visual analog scale (McCormack et al., 1988), global health score (Anderson et al., 2012), weight, height, body mass index (BMI), and the 28-joint disease activity score (DAS28) (Prevoo et al., 1995).

### C-reactive protein

High-sensitivity C-reactive protein (CRP) was measured in serum by latex-enhanced immunoturbidimetry on an automated Modular PPE Analyzer (Roche Diagnostics; Pepys and Hirschfield, 2003).

### Statistical analysis

Each analysis was performed in Stata version 14.2 (StataCorp, Texas, USA) using commands for structural equation modeling (SEM) with maximum-likelihood estimation. For each of the 16 different BP outcome variables we fitted a separate SEM model with MTX treatment status as a separate strata (grouping) variable. The endogenous (dependent) variables in each model were baseline and follow-up BP, and baseline and follow-up PWV. Exogenous (independent) variables in each model were age, gender, BMI, DAS28, serum CRP, and use of folic acid and prednisolone which were used as predictors of the baseline measures of BP and PWV. Measurement errors between BP and PWV were also allowed to co-vary at each time-point (Figure 1). The specific coefficients of interest were the effect of baseline PWV on follow-up BP (β_1_) and the effect of baseline BP on follow-up PWV (β_2_). Stratifying by MTX treatment status allowed separate coefficient estimates for each group of subjects and also calculation of the difference (and confidence intervals) for each coefficient between the 2 groups. We also performed a formal Wald test of group invariance in each coefficient to formally test whether the 2 estimates for each coefficient were the same for both groups. Model fit was determined using standard SEM measures: the chi-square (χ^2^) test of model fit vs. saturated fit for which non-significant values (*p* > 0.05) indicate acceptable fit, the comparative fit index (CFI) for which values >0.90 reflect acceptable model fit, and values >0.95 reflect excellent model fit, and the standardized root-mean-square residual (SRMSR) for which values of 0.05 or less reflect excellent model fit, while a value of <0.08 reflects a good fit. Given the 16 different measures of BP that were assessed, a Bonferroni correction was applied to the results and coefficients were only considered significant for *p* < 0.003. Similarly, we also calculated 99.7% confidence intervals (CI's).

## Results

### Descriptive statistics

Table 1 describes the baseline clinical and demographic characteristics of the patients in the MTX and the non-MTX groups. The mean (±SD) age of the population was 62 ± 13 and 78% were females. There were no differences between the non-MTX group of subjects and those taking MTX in terms of other medication use, chronic diseases and disease scores except for a significant difference for the presence of depression (38.9 vs. 14.6%, respectively, *p* = 0.04), the use of folic acid (5.6 vs. 75.6%; *p* < 0.001), the DAS28 (3.7 ± 1.0 vs. 2.6 ± 1.1, *p* < 0.001), and the Global Health score [median (IQR) 0.1 (0.38, 1.40) vs. 0.3 (0.04, 0.96); *p* = 0.03].

**Table 1:** Baseline clinical and demographic characteristics of the MTX and the non-MTX groups.

	**All subjects (*n* = 59)**	**Non-MTX group (*n* = 18)**	**MTX group (*n* = 41)**	***P*-value^a^**
Age (years)	62 ± 13	65 ± 13	61 ± 14	0.34
Females (%)	78.0	88.9	73.2	0.18
Body Mass Index (Kg/m^2^)	27.8 ± 6.2	28.6 ± 6.3	27.5 ± 6.2	0.55
Current smoking (%)	13.6	5.6	17.1	0.23
Hypertension (%)	35.6	38.9	34.2	0.73
Diabetes (%)	10.2	22.2	4.9	0.06
Dyslipidaemia (%)	30.5	38.9	26.8	0.75
Previous cardiovascular event (%)	8.5	22.2	2.4	0.03
Chronic kidney disease (%)	3.4	5.6	2.4	0.52
Liver disease (%)	3.4	5.6	2.4	0.52
Depression (%)	22.0	38.9	14.6	0.04
RA duration (years)	11 [4, 24]	15 [8, 27]	9 [3, 23]	0.21
MTX dose (mg/week)	14.3 ± 5.2	14.3 ± 5.2	–	–
DAS28 score	3.0 ± 1.2	3.7 ± 1.0	2.6 ± 1.1	<0.001
C-reactive protein (mg/L)	1.90 [0.57, 6.0]	1.95 [0.74, 4.1]	1.90 [0.57, 6.7]	0.76
Stanford HAQ score	0.5 [0.0, 1.5]	1.065 [0.38, 1.75]	0.25 [0.00, 1.13]	0.06
Pain visual analog score	0.76 [0.20, 1.34]	0.90 [0.40, 1.52]	0.70 [0.08, 1.24]	0.13
Global health score	0.58 [0.08, 1.30]	0.10 [0.38, 1.40]	0.30 [0.04, 0.96]	0.03
Hydroxychloroquine (%)	25.4	22.2	26.8	0.49
Leflunomide (%)	5.1	0.0	7.3	0.55
Sulfasalazine (%)	15.3	22.2	12.2	0.43
Abatacept (%)	3.4	5.6	2.4	0.52
Rituximab (%)	0.0	0.0	0.0	1.00
Tocilizumab (%)	8.5	22.2	2.4	0.03
Adalimumab (%)	6.8	5.6	7.3	0.64
Etanercept (%)	23.7	33.3	19.5	0.32
Certolizumab pegol (%)	1.7	2.4	0.0	1.00
Golimumab (%)	5.1	0.0	7.3	0.55
Prednisolone (%)	33.9	33.3	34.1	0.95
Prednisolone daily dose (mg)	5 [4.75, 7.50]	5.00 [4.0, 5.0]	[5.00 4.5, 10.0]	0.89
Ibuprofen (%)	6.8	0.0	9.8	0.30
Aspirin^*^ (%)	18.6	22.2	17.1	0.72
Antihypertensive drugs (%)	27.1	27.8	26.8	1.00
Folic acid (%)	54.2	5.6	75.6	<0.001
Fish oil (%)	37.3	27.8	41.5	0.39

*MTX, methotrexate; RA, rheumatoid arthritis; DAS, disease activity score 28; HAQ, health assessment questionnaire*.

* *Daily dose 100 mg in all patients*.

a *Using t-test, χ^2^-test or Fishers Exact as appropriate. Figures are Mean ± SD, median[25th percentile, 75th percentile], or n(%)*.

Table 2 describes the correlations, means and standard deviations for 24 h PWV, clinic peripheral SBP and 24 h peripheral SBP at the baseline and 8-month assessments (*n* = 59). Across the 2 time points, there was a slight increase in both clinic and ambulatory BP but there was no significant change in PWV. Amongst the 16 different measures of BP, pairwise Pearson correlations between measures of baseline SBP and follow-up PWV were moderate (*r* = 0.31–0.54), correlations between baseline DBP and follow-up PWV were weak (*r* = 0.06–0.20), correlations between baseline PWV and follow-up SBP were moderate (*r* = 0.40–0.55), correlations between baseline PWV and follow-up DBP were weak to moderate (*r* = −0.02 to 0.44), whilst correlations between baseline and follow-up measures of PWV were high (*r* = 0.91).

**Table 2:** Correlation matrix and descriptive statistics for PWV and clinic and 24 h peripheral SBP at baseline (0M) and 8-month (8M) assessments (*n* = 59).

	**Clinic SBP 0M**	**24 h PWV 0M**	**Clinic SBP 8M**	**24 h PWV 8M**
Clinic SBP 0M	1.00			
24 h PWV 0M	0.51	1.00		
Clinic SBP 8M	0.66	0.51	1.00	
24 h PWV 8M	0.54	0.91	0.55	1.00
*N*	59	59	59	59
Mean	126.1	9.10	127.7	9.08
SD	17.5	2.01	18.1	2.18
	**24 h SBP 0M**	**24 h PWV 0M**	**24 h SBP 8M**	**24 h PWV 8M**
Clinic SBP 0M	1.00			
24 h PWV 0M	0.53	1.00		
Clinic SBP 8M	0.56	0.40	1.00	
24 h PWV 8M	0.43	0.91	0.44	1.00
*N*	59	59	59	59
Mean	126.5	9.10	107.9	9.08
SD	12.7	2.01	15.41	2.18

*SD, standard deviation*.

### Effect of PWV on BP (β_1_ path coefficients)

**Figure 2:**
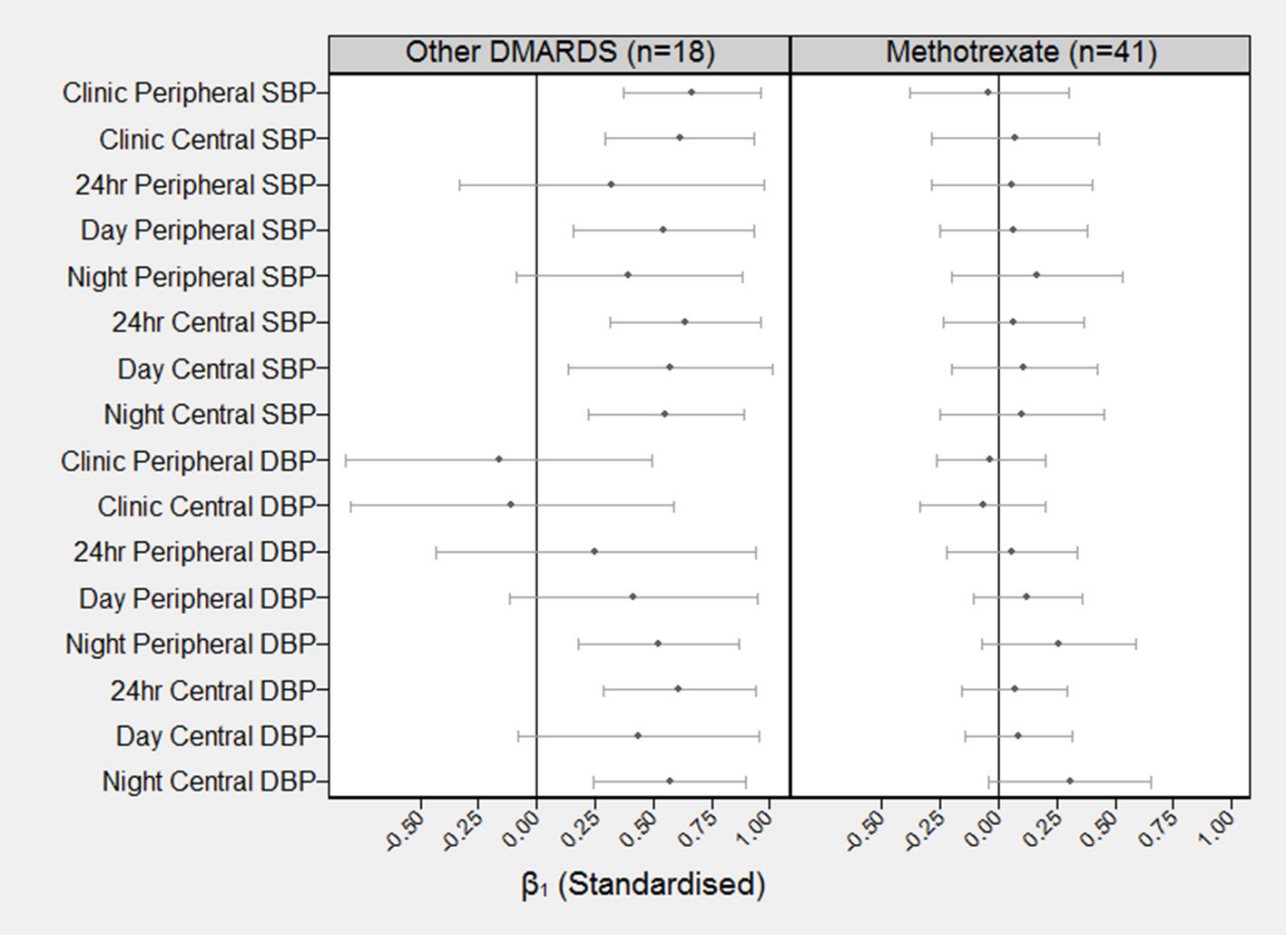
Standardized coefficients (and 99.7% confidence intervals) describing the correlation between baseline PWV and BP at follow-up BP (β_1_). The standardized β_1_ can be interpreted as the number of SD increases in BP at 8 months for each one SD increase in PWV at baseline. For example, the standardized β_1_ coefficient for baseline PWV on clinic peripheral SBP for the other DMARDS group is β_1_ = 0.66 (99.7% CI = 0.37–0.96).

Figure 2 shows the standardized estimates with 99.7% CI's for β_1_ by DMARD treatment regime. For subjects in the non-MTX group, out of the 8 measures of SBP, all except 24 h peripheral SBP and night-time peripheral SBP showed a significant effect of baseline PWV on follow-up BP (standardized β, range = 0.54–0.66, *p* < 0.003 for each). Out of the 8 measures of DBP, 3 showed a significant effect of baseline PWV on follow-up BP (standardized β, range = 0.54–0.66, *p* < 0.003 for each) including night-time peripheral, 24-h central and night-time central DBP. There were no significant effects of baseline PWV on measures of either SBP or DBP amongst subjects using MTX.

### Effect of BP on PWV (β_2_ path coefficients)

**Figure 3:**
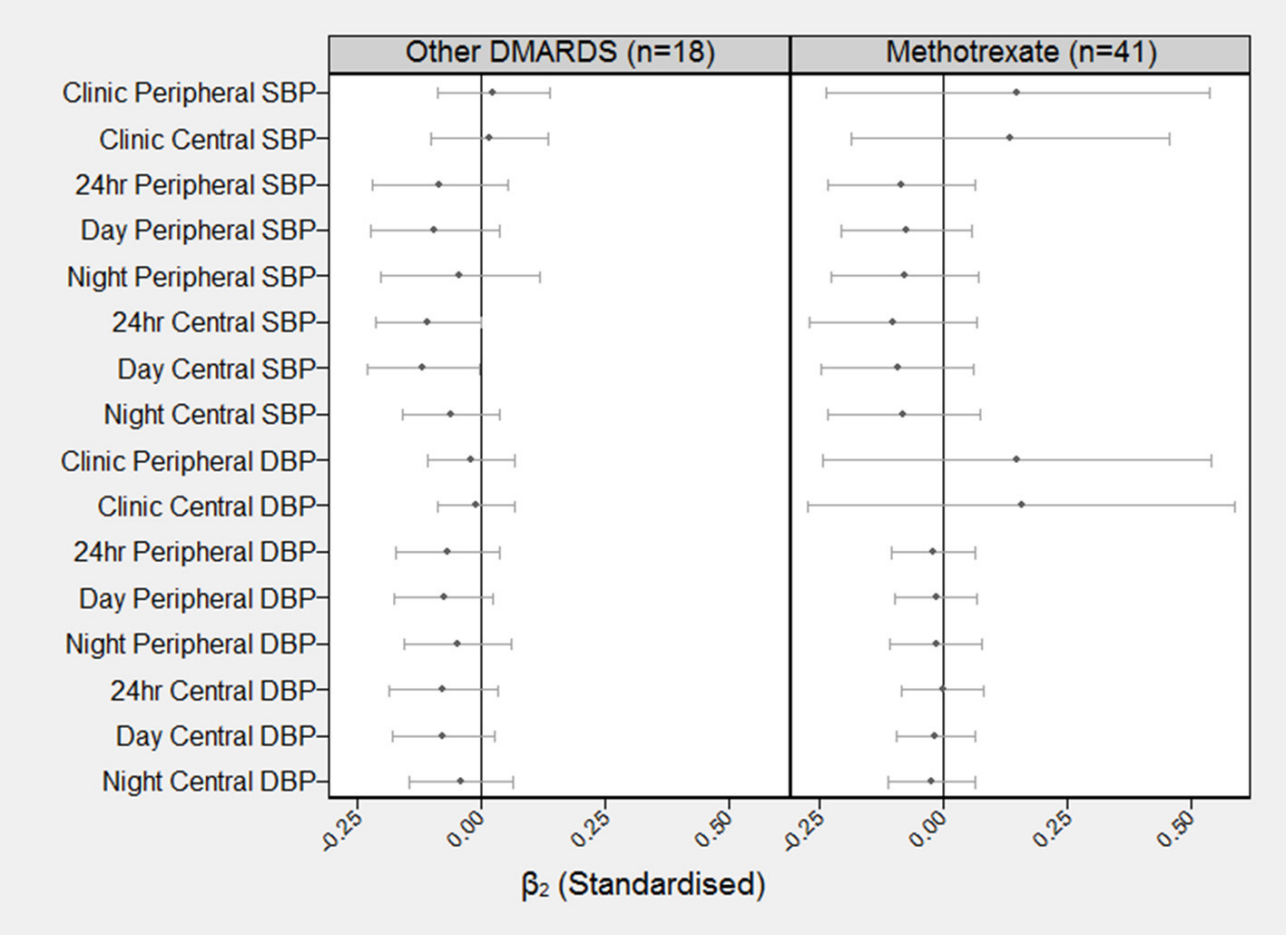
Standardized coefficients (and 99.7% confidence intervals) describing the correlation between baseline BP at follow-up PWV (β_2_). The standardized β_2_ can be interpreted as the number of SD increases in PWV at 8 months for each one SD increase in BP at baseline. For example, the standardized β_2_ coefficient for baseline BP on clinic peripheral PWV for the other DMARDS group is β_2_ = 0.02 (99.7% CI = −0.09–0.14).

Figure 3 shows the standardized estimates with 99.7% CI's for β_2_ by type of DMARD. For both those subjects in the non-MTX group and those in the MTX group, there were no significant effects of baseline SBP or baseline DBP, on follow-up PWV.

### Difference in path coefficients between groups

**Figure 4:**
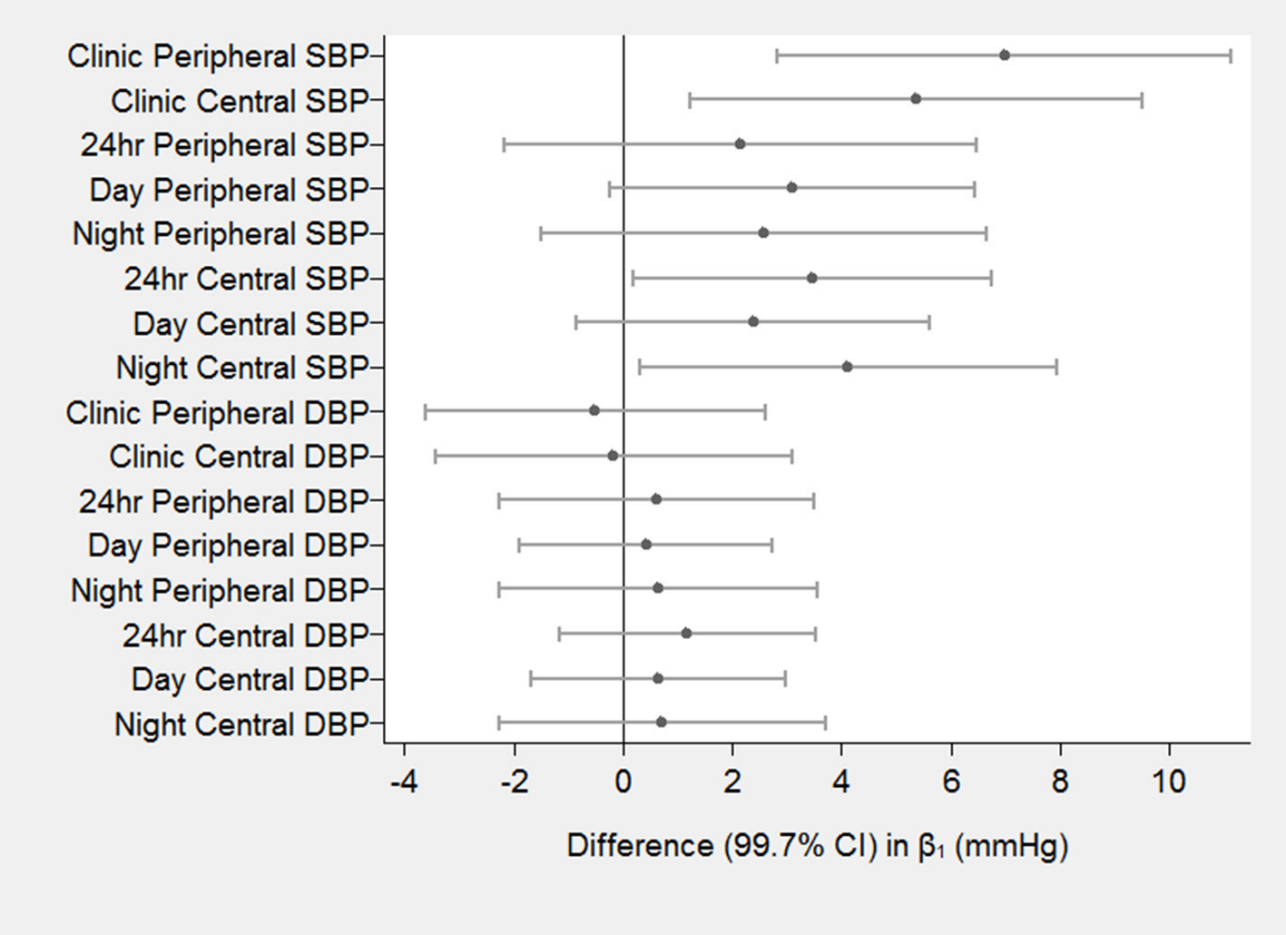
Differences in the effects of baseline PWV on follow-up BP between DMARDS group and MTX group. The estimated effects (β) are the differences in increased blood pressure (in mmHg) at 8 months for a 1 m/s higher baseline PWV for DMARDS vs. MTX.

Figure 4 shows the difference in the estimated effects of baseline PWV on increases in BP at 8 months (β_1_) for the different treatments (DMARDs vs. MTX). There was a significant difference between groups in the estimated effects for clinic peripheral SBP, clinic central SBP, 24 h central SBP and night central SBP (*p* < 0.003 for each). The largest difference in the effect occurred for clinic peripheral SBP (β = 7.0, 95% CI = 2.8–11.1 mmHg per 1 m/s higher baseline PWV; *p* < 0.001). There were no significant differences between groups in the effects of baseline PWV on any of the measures of follow-up DBP. There were also no significant differences between groups in β_2_ (effect of baseline BP on follow-up PWV) for any of the 16 measures of BP. The Wald test for group invariance in the estimated coefficients between the 2 groups showed a significant difference in the β_1_ coefficient for clinic peripheral SBP (*p* = 0.0004), clinic central SBP (*p* = 0.006), day-time peripheral SBP (*p* = 0.014), 24 h central SBP (*p* = 0.005), day-time central SBP (*p* = 0.045), and night-time central SBP (*p* = 0.013). There was also a significant difference in the β_2_ coefficient for clinic peripheral DBP (*p* = 0.043) and clinic central DBP (*p* = 0.046; Figures 3, 4).

### Model fit statistics

Table 3 describes the various indices of model fit for each of the 16 measures of BP. Overall, the indices indicated excellent model fit with the mean CFI = 0.956 and the mean SRMSR = 0.046. The mean χ^2^ value for the fitted model vs. the saturated model was χ^2^ = 44.0 (28 df) (*p* = 0.028) indicating acceptable fit.

**Table 3:** Indices of model fit for each of the 16 blood pressure models.

**Blood pressure variable**	**χ^2^ test (28 df)^a^**	***p*-value**	**CFI^b^**	**SRMSR^v^**
Clinic peripheral SBP	44.8	0.023	0.95	0.05
Clinic central SBP	38.9	0.082	0.97	0.05
24 h peripheral SBP	36.8	0.122	0.98	0.03
Day peripheral SBP	36.1	0.141	0.98	0.04
Night peripheral SBP	47.4	0.012	0.95	0.04
24 h central SBP	35.8	0.147	0.98	0.04
Day central SBP	34.3	0.190	0.98	0.04
Night central SBP	47.4	0.013	0.95	0.05
Clinic peripheral DBP	32.6	0.253	0.99	0.05
Clinic central DBP	37.1	0.116	0.97	0.06
24 h peripheral DBP	49.6	0.007	0.93	0.05
Day peripheral DBP	60.7	<0.001	0.91	0.06
Night peripheral DBP	51.1	0.005	0.93	0.04
24 h central DBP	55.8	0.001	0.93	0.05
Day central DBP	47.2	0.013	0.95	0.05
Night central DBP	47.9	0.011	0.94	0.04

a Chi-squared test of fitted model vs. saturated fit with 28 degrees of freedom;

b CFI, Comparative fit index;

*^c^SRMSR, Standardized Root Mean Square Residual*.

### Changes in heart rate and CRP

Table 4 displays the changes between baseline and follow-up in average 24-h heart rate and log-transformed CRP for each of the 2 groups, as well as the associated differences in these changes between groups. There were no significant differences between baseline and follow-up for each group, and no significant difference in the change between groups.

**Table 4:** Twenty-four hour heart rate and CRP values at baseline and follow-up for each group and estimated changes.

	**24-h heart rate**			**Change MTX vs. Non-MTX (*p*-value)^b^**
	**0 months**	**8 months**	**Change^a^**	
Non-MTX (*n* = 18)	68.9 ± 10.7	69.1 ± 8.6	0.2 ± 1.7 (*p* = 0.90)	
MTX (*n* = 41)	71.2 ± 9.4	70.2 ± 9.6	−1.1 ± 1.1 (*p* = 0.35)	−1.30 ± 2.07 (0.53)
	**Log CRP**			
	**0 months**	**8 months**	**Change**^a^	
Non-MTX (*n* = 1 8)	0.57 ± 1.31	0.40 ± 1.31	−0.21 ± 0.36 (*p* = 0.56)	
MTX (*n* = 41)	0.79 ± 1.67	0.49 ± 1.61	−0.30 ± 0.23 (*p* = 0.19)	−0.09 ± 0.43 (*p* = 0.83)

a *Adjusted marginal means using mixed effects linear regression model*.

b *From MTX × visit interaction term*.

## Discussion

In this repeat cross-sectional study of patients with stable RA, we examined the strength of the temporal relationships between arterial stiffness (PWV) and follow-up BP and between baseline BP and follow-up PWV. We observed evidence for a causal effect of arterial stiffness on increased SBP in subjects not treated with MTX. The effects were seen for both central and peripheral and for clinic and ambulatory SBP. The effects were similar during the day and night and we also observed the same effect of PWV on night-time and 24 h central DBP amongst subjects not taking MTX. By contrast, none of the causal effects of increased stiffness on BP were observed amongst subjects treated with MTX medication. Since the differences in causal effects were predominantly related to systolic blood pressure, rather than diastolic pressure, this suggests the possible involvement of effects on stroke volume. Since we did not measure stroke volume directly, further studies are required to determine whether this parameter might explain, at least in part, the observed between-group differences.

Since inflammation is believed to have a primary influence in the pathophysiology of increased arterial stiffness (Jain et al., 2014), studying a population known to have high underlying levels of inflammation, and associated cardiovascular risk, provides a valuable opportunity to examine the extent to which inflammation and specific DMARDs may influence the stiffness-BP relationship. The 2 groups of subjects within our own study had similar values of PWV at baseline, which were in line with values normally seen for their age. Our results therefore suggest that arterial stiffness related increases in BP were prevented in those using MTX. If this finding were also to be observed in individuals without RA, it would support the idea that the effects of increased arterial stiffness may partly be due to underlying inflammation, and that MTX acts to disrupt these specific pathways more effectively than other DMARDs. Furthermore, MTX appears to exert additional vasculoprotective effects independent of reduced inflammation, through the accumulation of adenosine, a vasodilator, BP lowering, and stimulating nitric-oxide (NO) synthesis agent, and stimulation of 5′ AMP-activated protein kinase (AMPK) (Costa and Biaggioni, 1998; Tian and Cronstein, 2007; Schneider et al., 2015; Thornton et al., 2016). In this study we observed non-significant reductions in CRP, a measure of systemic inflammation, of ~37% in each group, suggesting that systemic inflammation was not a major explanatory factor of the observed causal effects.

Our results may not be generalizable to all individuals with RA, particularly younger patients. Our cohort was typical of the RA population and consisted of mostly middle-aged to older patients. Recently, the Bogalusa study, a 7 year longitudinal cohort study in a younger population of adults, used similar methods to ours to examine the dominant temporal relationship between arterial stiffness and BP. In this younger cohort they reported that elevated BP precedes increased arterial stiffness rather than vice-versa (Chen et al., 2016) and concluded that amongst younger adults the arterial wall may not yet be stiff enough to significantly influence BP.

Our study had a number of strengths. Firstly we used cross-lagged path analysis, a specific form of path analysis that simultaneously examines reciprocal, longitudinal relationships among a set of inter-correlated variables and allows better determination of causal relationships (Selig and Little, 2012). Overall, our models provided excellent levels of fit indicating that the proposed causal pathways were feasible for the given data. In addition we used a wide variety of BP measures including 24 h BP monitoring with repeat measurements every 20 min during the day and every 30 min during the night. This reduces measurement error and increases statistical power. We also collected clinical data on a large number of potential confounders and were able to demonstrate similarity between groups except for a few variables including DAS28 and use of folic acid that were adjusted for in our analysis.

There are several limitations to our study. The duration of follow-up in our study was relatively short with a mean of 8 months. Most of the evidence from other studies have been derived from cohorts with between 4 and 8 years average follow-up in which larger effects are likely to occur (Chen et al., 2016). However, the strength of the associations observed in these studies and our own study were similar. In addition, our sample size was relatively small and so we may have been underpowered to detect some of the smaller effects that were observed. Although, characteristic of a RA population, the study population was also fairly heterogeneous with some subjects receiving BP medications as well as different anti-inflammatory medications for the treatment of their RA. Although, we adjusted for the use of prednisolone, a more homogeneously treated population would allow us to better explore the associations without the risk of selection bias. Finally, although we tried to reduce the possibility of confounding and also used a structural equation modeling approach to better identify the most likely direction of causality, the results may still be influenced by residual confounding and reverse causality since not all subjects were free of cardiovascular disease at baseline. A fully randomized controlled trial in a disease free non-hypertensive population is required to determine both the true causal effects of PWV on BP and also whether MTX use can inhibit the damaging effects of increased stiffness.

In summary we have demonstrated that although elevated arterial stiffening preceded increases in BP in subjects with RA, these effects did not occur amongst those patients using MTX medication. The beneficial effects were seen mostly in SBP but were also apparent to some degree with DBP, particularly with regards to the more accurate assessment of 24-h blood pressures. These findings suggest MTX may confer a protective effect against stiffness mediated increases in BP in patients with RA.

## Author contributions

RW performed the analysis, helped conceive the study design, provided Ph.D. supervision, performed the first draft of the manuscript and reviewed later versions. LB collected and formatted the data for the study as part of her Ph.D., performed ethics applications, obtained consent from patients and reviewed several versions of the manuscript. MS was involved in all aspects of the study design, provided clinical patients for the study, and reviewed the manuscript before submission. AM was involved in all aspects of the study design, Ph.D. supervision, helped draft the first version of the manuscript and provided several revisions of the manuscript.

### Conflict of interest statement

The authors declare that the research was conducted in the absence of any commercial or financial relationships that could be construed as a potential conflict of interest.
